# Partial anomalous pulmonary venous connection: how many vs. how much

**DOI:** 10.1186/1532-429X-11-S1-P274

**Published:** 2009-01-28

**Authors:** Brian Grant, Shi-Joon Yoo, Mike T Seed, Lars Grosse-Wortmann

**Affiliations:** Hospital for Sick Children, Toronto, ON Canada

**Keywords:** Pulmonary Vein, Body Surface Area, Lung Volume, Sinus Venosus, Total Lung Volume

## Introduction

The diagnosis of partial anomalous pulmonary venous connection (PAPVC) is usually made by echocardiography. However, complete anatomical delineation is often difficult. In addition, there has been controversy regarding which patients require surgical intervention. Magnetic resonance imaging (MRI) can provide comprehensive information on precise anatomy, systemic-to-pulmonary shunt (Qp:Qs) and ventricular volumes of patients with PAPVC.

## Purpose

To (i) quantify the degree of left to right shunting, (ii) describe the distribution of blood flow to the right and the left lung, (iii) assess the relationship between the number of anomalously draining veins and Qp:Qs, (iv) assess the affect of other associated defects on Qp:Qs.

## Methods

We retrospectively reviewed MRIs performed on patients with PAPVC in our institution over the last 5 years. All patients were assessed with cine imaging, contrast-enhanced angiography, and phase-contrast velocity mapping. The number of anomalous veins was recorded, and the proportion of blood flow from the anomalously draining vein relative to the total flow to that lung was measured. The proportion of anomalously drained lung volume relative to the total lung volume was estimated. Flow volumes indexed to body surface area (BSA) through the right and left pulmonary arteries and the ascending aorta were used for calculation of Qp:Qs. Ventricular end diastolic and end systolic volumes were measured and indexed to BSA.

## Results

There were nineteen patients (13 male, mean age 11.1 ± 5.8 years) with a diagnosis of PAPVC that had undergone an MRI study. Four patients were a new diagnosis made by MRI, and not detected by echocardiography. In a further 3 cases, echocardiographic suspicion was confirmed. Twelve patients had right PAPVC, 6 had PAPVC of the left upper pulmonary vein, and 1 patient had right-sided unilateral total anomalous pulmonary venous drainage. In 8 patients, there was an associated sinus venosus defect (SVASD). Mean Qp:Qs was 1.75 ± 0.4, and increased to 2.5 ± 0.6 in the presence of a SVASD. Qp:Qs correlated with right ventricular end diastolic volume index (ρ = 0.54; p = 0.02) and the proportion of anomalous veins (ρ = 0.77; p = 0.01). We found no increased differential pulmonary arterial blood flow to lungs with PAPVC from normal (p = 0.001). In individual cases, a high Qp:Qs in the setting of a single anomalously draining pulmonary vein could be explained by angiographic assessment of the estimated volume of lung drained by the vein.

## Conclusion

We believe that for the diagnosis of PAPVC, MRI not only allows a complete definition of the anatomy, especially in the setting of SVASD, it also details the ensuing hemodynamic consequences. Both often provide important additional information in the indication of surgery and procedure planning.

